# Should serum creatinine kinase levels and an ECG be routinely obtained in low voltage electrical burn injuries?

**DOI:** 10.1186/1757-7241-21-S1-S20

**Published:** 2013-05-28

**Authors:** JR Pallett, M Tunnicliff, JW Keep

**Affiliations:** 1 Kings College Hospital, London, UK

## Background

Initial excess of creatinine kinase (CK) has been shown to correlate with extent of muscle damage in electrical burns.[1] High voltage burns leading to extensive muscle damage, compartment syndrome and rhabodmyolysis is clinically apparent at an early stage. However, the frequency of these injuries is rare. Low voltage (<1000V) injuries from domestic appliances are far more common with few if any clinical signs. In order to develop a local guideline, the current practise of investigations ordered in our department was analysed.

## Methods

A retrospective cohort study of all electrical burn injuries at King’s College Hospital Major Trauma Centre over a 4 year period was analysed. Mechanism of injury, serum CK level, ECG findings and outcome were recorded.

## Results

57 electrical burn injuries were identified from the trauma registry between August 2008 and October 2012. Mechanism of injury was recorded in 40 cases. All were from domestic appliances (< 1000V). A mean age of 34.7yrs (range 10 – 80) was observed. Serum CK levels were taken in 32 cases. In 15 cases, levels were elevated (> 150IU/L) ranging from 164-697. In only 1 of these cases was a superficial burn injury observed. Examination was normal in all the other patients. All patients with abnormal CK levels were discharged from the ED without influencing management and no serial CK levels were taken. 26 cases had documented evidence of an ECG being performed, none of which were abnormal. 2 patients with paraesthesia were referred for outpatient electromyography and nerve conduction studies which subsequently showed no abnormality. 1 case was referred to the regional burns unit. The remainder were all discharged directly from the ED.

## Conclusions

For electrical injuries sustained from domestic appliances, in the absence of clinical signs of tissue damage, routine measurement of CK and ECG recording does not appear to influence management and little evidence is available for their current routine use in these patients. Safe levels at which patients can be discharged with abnormal CK levels and need for serial CK level measurements needs to be studied further.
